# Death Anxiety and Death Education Needs Among Community‐Dwelling Older Adults in Western China: A Preliminary Cross‐Sectional Survey From the Perspective of Palliative Care Promotion

**DOI:** 10.1002/nop2.70962

**Published:** 2026-11-07

**Authors:** Yan Wang, Xiaolan Guo, Liqun Huang, Sha Li, Yuchen Wang

**Affiliations:** ^1^ Shaanxi University of Chinese Medicine Xianyang Shaanxi China; ^2^ The Second Affiliated Hospital of Shaanxi University of Chinese Medicine Xianyang Shaanxi China

**Keywords:** ‘Four‐Step’ death education model, community care, community‐dwelling older adults, death anxiety, death education, palliative care

## Abstract

**Aim:**

To describe death anxiety and death education needs among community‐dwelling older adults in Xianyang, Shaanxi Province, China; to examine their association; and to identify factors independently associated with death education needs as a preliminary needs assessment for palliative care promotion.

**Design:**

Descriptive, cross‐sectional survey.

**Methods:**

A convenience sample of 267 community‐dwelling older adults was recruited. Data were collected using a general information questionnaire, the Templer Death Anxiety Scale (T‐DAS) and an adapted Death Education Needs Scale. Both online (WQStar) and face‐to‐face surveys were used. Descriptive statistics, univariate analysis, Spearman correlation and multiple linear regression were performed.

**Results:**

Most participants (91.0%) had no prior exposure to death education. The median T‐DAS score was 5.00 (3.00, 6.00), and the median death education needs score was 39.00 (34.00, 44.00). Death anxiety was weakly positively associated with death education needs. Only the highest monthly income category (≥ 4000 yuan) was associated with higher death education needs; educational level was not independently associated in the multivariable model. Lectures and films/documentaries were the most frequently preferred formats. The adapted Death Education Needs Scale showed preliminary internal‐consistency and item‐structure evidence only.

**Conclusion:**

Community‐dwelling older adults in this regional sample reported low prior exposure to death education and favourable attitudes towards its value. Death anxiety was weakly positively associated with death education needs. Monthly income was associated with needs. The adapted scale requires further validation. These preliminary findings provide regional data for hypothesis generation; larger‐scale, more rigorously designed longitudinal or intervention studies are needed.

**Implications for the Profession and/or Patient Care:**

Community nurses should avoid directly discussing death with older adults who are uncomfortable with the topic; they can use softer questions, listen and identify individual information preferences. For older adults who are open to discussion, nurses can provide practical information and refer to palliative care or advance care planning when wanted. The adapted scale measures attitudes towards death education, not individual unmet need. Because this cross‐sectional study is descriptive only, it cannot provide guidance on any specific effective intervention.

**Impact:**

This study provides regional and incremental evidence from western China. Previous research has already reported substantial death education demand among Chinese community‐dwelling older adults. The present findings may serve as a reference for hypothesis generation in community‐based end‐of‐life care and nursing practice.

**Reporting Method:**

Reported according to the STROBE checklist for cross‐sectional studies.

**Patient or Public Contribution:**

No patient or public contribution.

According to data from the National Bureau of Statistics, China's population aged 60 and older exceeded 280 million in 2023, accounting for 19.8% of the total population. The aging trend is characterized by accelerated progression, substantial demographic scale and a marked increase in the proportion of the oldest‐old. Compounded by high rates of chronic diseases and multimorbidity, older adults face growing risks related to aging, illness and mortality (Bao et al. 2022). Influenced by the traditional cultural notion of ‘speaking of life but avoiding mention of death’, however, death has long been avoided and neglected in society, contributing to public misconceptions and negative attitudes that perpetuate death anxiety (Song et al. 2019). Such negative emotional experiences: fear, worry and psychological anguish, can impair older adults' mental health, quality of life and end‐of‐life adjustment. Persistent death anxiety has been associated with reduced subjective well‐being, depression, loneliness and poorer chronic disease management and palliative care compliance (Lin et al. 2024).

As a critical intervention, death education systematically provides knowledge about mortality, cultivates coping strategies and fosters healthy perspectives, helping individuals reconcile with end‐of‐life processes and grief (Hu 2022). Internationally, death education has developed into a mature field with comprehensive curricula and social support networks (Seckin et al. 2025). In China, however, it started relatively late. Some universities offer standalone courses to help students establish values regarding life (Hu 2015), and medical institutions use palliative or hospice care to reduce physical and mental suffering and improve the dying experience (Liu et al. 2022; Jiang et al. 2019). Nevertheless, these models remain limited, and development has been slow under traditional cultural influences. Death education still occupies a marginalized position, and a systematic, socialized, full‐life‐cycle educational ecosystem has not yet been established (Chen 2023; Xu et al. 2020).

In model development, Huang et al. (2018) evidence‐based ‘Four‐Step’ approach, validated among medical students, provides a foundational framework. The model includes four sequential steps: (1) cognitive awakening: recognizing death as a natural part of life and understanding life's finitude; (2) emotional expression: identifying and expressing fear, anxiety, grief and other death‐related emotions; (3) meaning reconstruction: using life review, narrative sharing and group discussion to reconstruct personal meaning and values concerning life and death; and (4) behavioural preparation: supporting end‐of‐life communication, advance care planning and coping with bereavement. For community‐dwelling older adults, this model suggests beginning with less threatening topics and gradually moving towards more sensitive end‐of‐life issues. Zhang et al. (2022) further showed that progressive education can improve older adults' death attitudes and existential meaning. Yet application gaps remain because older adults have distinct physiological conditions, cultural contexts and perceptions of mortality that require appropriate content and delivery formats (Subiyanto and Susilo 2024).

Death education demand among community‐dwelling older adults in China has been previously reported, but evidence from western China remains limited. Therefore, this study aimed to describe death anxiety and death education needs among community‐dwelling older adults in Xianyang, Shaanxi Province, and to examine factors associated with death education needs. Three research questions were addressed: (1) What are the levels of death anxiety and death education needs among community‐dwelling older adults? (2) Is death anxiety associated with death education needs? (3) Which sociodemographic and health‐related characteristics are independently associated with death education needs? We hypothesized that death anxiety would be positively associated with death education needs, and that monthly income and educational level would be associated with needs in univariate analysis. We explored whether either remained independently associated in multivariable analysis.

## 1 Subjects and Methods

### 1.1 Study Design and Participants

This study was reported according to the STROBE checklist for cross‐sectional studies. A cross‐sectional survey was conducted among community‐dwelling older adults in Xianyang, Shaanxi Province, China.

### 1.2 Study Population

Convenience sampling was used to select older adult residents in communities in Xianyang City, Shaanxi Province, as the study population from December 2025 to March 2026. Inclusion criteria: (1) age ≥ 60 years; (2) alert and able to answer questions independently; (3) willing to participate in the study and sign an informed consent form. Exclusion criteria: (1) no fixed residence; (2) individuals with mental disorders or those unable to communicate effectively.

Based on the number of independent variables, the sample size was calculated as 10–15 times the number of variables. Accounting for a 20% invalid questionnaire rate, at least 163–244 participants were required. A total of 267 valid questionnaires were finally included, which met the sample size requirement. Before the commencement of this study, approval was obtained from the Ethics Committee of the Second Affiliated Hospital of Shaanxi University of Chinese Medicine (LW2026002‐1).

### 1.3 Research Instruments

#### 1.3.1 Demographic Data Survey

A self‐designed questionnaire was used, covering gender, age, educational level, religious beliefs, monthly income, marital status, history of health‐related distress, history of life‐threatening events, history of bereavement, prior exposure to death education and preferred methods for conducting death education.

#### 1.3.2 Death Anxiety Scale

The Chinese version of the Death Anxiety Scale (Yang 2011) (Templer's Death Anxiety Scale, T‐DAS), cross‐culturally adapted by Chinese scholar Yang Hong, was selected. The Chinese version of the T‐DAS comprises four dimensions: Emotional (6 items), Stress and Distress (4 items), Sense of Time (2 items) and Cognitive (3 items), totalling 15 items. Each item is scored as 1 or 0, with items 2, 3, 5, 6, 7 and 15 scored negatively. The total scale score ranges from 0 to 15. A higher score indicates a higher level of anxiety. The scale's overall Cronbach's *α* coefficient is 0.71, indicating good reliability and validity.

#### 1.3.3 Adapted Death Education Needs Scale

The original Death Education Needs Scale was developed by Yan (2002) to assess death education needs among nursing college students in Taiwan, China. In this study, it was adapted for community‐dwelling older adults with the developers' conceptual framework as a reference. First, a panel of 4 experts in geriatric nursing and palliative care reviewed the item wording. They suggested revising the context, language, and direct or sensitive death‐related expressions to improve cultural appropriateness and acceptability for older adults. For example, the phrase ‘facing death’ was revised to ‘facing the final stage of life’, and ‘preparation for death’ was revised to ‘preparation for the terminal stage of life’. These euphemistic expressions were intended to preserve the original meaning while improving cultural acceptability. Based on expert feedback, all 10 items were revised to improve their suitability for completion by community‐dwelling older adults. Second, a small‐scale pilot test was conducted among 15 community‐dwelling older adults to assess readability, comprehension and completion time. Based on their feedback, the wording of three items was further refined. The average completion time was 3–5 min. No items were removed during this process. The final adapted scale contained 10 items rated on a 5‐point Likert scale (1 = strongly disagree to 5 = strongly agree), with a total score of 10–50 A higher score indicates greater death education needs. The total score should be interpreted as perceived benefits, social importance and attitudes towards death education, not as a definitive measure of unmet individual educational need. The internal consistency and factor structure of the adapted scale were evaluated using Cronbach's alpha and exploratory factor analysis in the final sample; detailed results are presented in the Section 2.

### 1.4 Research Methods

A mixed online and face‐to‐face survey was conducted. Given that many older adults have limited access to or familiarity with electronic devices, face‐to‐face administration was used alongside the online survey to improve accessibility. The questionnaire contained 36 items across four pages: Page 1, welcome message and survey instructions; Page 2, general demographic information (11 items); Page 3, Death Anxiety Scale (15 items); Page 4, Death Education Needs Scale (10 items). The average completion time was approximately 8–12 min.

#### 1.4.1 Online Survey

The online questionnaire was developed using WQStar; participants could navigate back to previous pages to modify their answers. All items were set as required, so skipping was not permitted. No financial or material incentives were provided. Duplicate submissions were prevented by limiting one submission per WQStar account. The research team did not access, export, or analyse account identifiers, IP addresses, or other potentially identifying metadata. Data were exported with anonymous study IDs only. Before formal data collection, two researchers tested the questionnaire on desktop and mobile devices for display, skip logic and data export. A pilot test was conducted among 10 older adults to assess readability and completion time. Recruitment was conducted through community health service centres, senior activity centres, WeChat groups, local social media groups and community bulletin boards. The invitation text was: ‘Hello! We are conducting an anonymous survey on death anxiety and death education needs among community‐dwelling older adults. It will take approximately 8–12 minutes. Your participation is voluntary, and you may withdraw at any time. All information will be used only for research purposes and kept strictly confidential. Thank you for your support!’.

#### 1.4.2 Face‐to‐Face Survey

Older adults were approached in community settings and guided one‐on‐one through the questionnaire. Questionnaires were collected on‐site and checked for omissions; missing responses were supplemented as needed. Questionnaires with missing or incorrect entries, or those with the same option selected for all items, were considered invalid. Questionnaires were checked for omissions. When an item was missing, the researcher reminded the participant and invited the participant to complete it themselves. Research staff did not provide answers, suggest responses or complete items on participants' behalf. During administration of death‐related questions, participants could pause or withdraw at any time. If a participant showed distress, the researcher paused the survey, provided reassurance and offered referral information for community psychological, community health or medical support. Participation continued only if the participant wished to continue.

### 1.5 Statistical Methods

Statistical analysis was performed using SPSS 27.0. Categorical data were described using frequencies and percentages. Continuous data with skewed distribution were presented as median (IQR). Intergroup comparisons were performed using the Mann–Whitney *U* test or the Kruskal–Wallis *H* test. Multivariate regression analysis was used to examine associated factors. Differences were considered statistically significant when *p* < 0.05.

Covariates were selected based on previous research evidence and theoretical considerations (Zhang et al. 2025; Lei et al. 2022; Fortner and Neimeyer 2007; Wang et al. 2024; Smith and Harkness 2011), the main included educational level, monthly income, age, health status, bereavement experience and marital status. Because these factors have been linked to death anxiety, death education demand or end‐of‐life care needs.

A sensitivity power analysis using G*Power 3.1.9.2 was planned based on the final sample size and the number of predictors retained in the final multivariable model, to estimate the minimum detectable effect size. In addition, to examine the potential influence of survey mode, a separate sensitivity analysis was planned by adding survey mode (online vs. face‐to‐face) as a covariate to the regression model. The detailed results of these sensitivity analyses are reported in the Section 2.

## 2 Results

### 2.1 Demographic Characteristics

For the online survey, 63 individuals started the survey, 63 completed it, and 63 valid questionnaires were included (valid completion rate = 100.0%). For the face‐to‐face survey, 210 older adults were approached, 204 completed the questionnaire and 204 valid questionnaires were included (valid response rate = 97.1%). Overall, 267 valid questionnaires were included.

A total of 267 community‐dwelling older adults were included in the study, comprising 91 men (34.1%) and 176 women (65.9%); the majority were aged 60–65 years (52.1%); the majority had a high school education (40.4%); 89.5% had no religious affiliation; the largest group had a monthly income of ≤ 2000 yuan (29.2%); 84.3% were married with a living spouse; 41.6% had chronic diseases; 81.6% had not experienced a life‐threatening event; 59.6% had experienced the loss of a loved one; 91.0% had never been exposed to death education; among preferred methods of death education, ‘lectures’ were the most common (27.2%), followed by ‘watching movies, documentaries, etc.’ (20.3%), ‘reading books, magazines, etc.’ (13.5%) and ‘group discussions and experience sharing’ (11.6%). See Table 1 for details.

**TABLE 1:** General characteristics of community‐dwelling older adults (*n* = 267).

Category	Subcategory	Number	Percentage (%)
Gender	Male	91	34.1
	Female	176	65.9
Age	60–65	139	52.1
	66–70	90	33.7
	75 and above	38	14.2
Educational level	Elementary school and below	30	11.2
	Junior High	80	30.0
	High School	108	40.4
	College/University and above	49	18.4
Religious beliefs	Yes	28	10.5
	No	239	89.5
Monthly income	≤ 2000	78	29.2
	2000–2999	73	27.3
	3000–3999	52	19.5
	≥ 4000	64	24.0
Marital status	Unmarried	4	1.5
	Married with spouse alive	225	84.3
	Divorced	9	3.3
	Widowed	29	10.9
Health conditions	Chronic diseases	111	41.6
	Malignant tumours	16	6.0
	Traumatic diseases	7	2.6
	No	133	49.8
Life‐threatening events	Yes	49	18.4
	No	218	81.6
Experience of bereavement	Yes	159	59.6
	No	108	40.4
Exposure to death education	Yes	24	9.0
	No	243	91.0
Preferred methods of death education (multiple choices)	Lectures	164	27.2
	Reading books, magazines, etc.	81	13.5
	‘Simulated farewell’ activities	49	8.1
	Watching movies, documentaries, etc.	122	20.3
	Group discussions and experience sharing	70	11.6
	Field trips	38	6.3
	Creative writing or arts	60	10.0
	Other	18	3.0

*Note:* For ‘Preferred methods of death education’, participants could select multiple options. Percentages for this item are therefore based on the total number of responses (*n* = 602) rather than the number of participants.

### 2.2 Preliminary Internal Consistency and Item‐Structure Evidence for the Adapted Scale

The internal consistency of the adapted Death Education Needs Scale was examined using Cronbach's alpha based on the final sample of 267 community‐dwelling older adults. The total‐scale Cronbach's alpha was 0.938. Corrected item‐total correlations ranged from 0.679 to 0.783, and no item deletion would improve the overall alpha. A principal component analysis was used only as a preliminary item‐structure check; it is not equivalent to exploratory common‐factor analysis. The KMO value was 0.938, and Bartlett's test of sphericity was significant (*χ* ^2^ = 1881.957, df = 45, *p* < 0.001). One factor with an eigenvalue > 1 was extracted, explaining 64.466% of the total variance. All 10 items loaded on this single factor, with factor loadings ranging from 0.739 to 0.832. These findings provide preliminary internal‐consistency and item‐structure evidence only. The scale requires further validation in independent samples.

### 2.3 Death Anxiety Scores Among Community‐Dwelling Older Adults

The total death anxiety score for community‐dwelling older adults was 5.00 (3.00, 6.00), among the subscales, the ‘Emotions’ subscale had the highest score at 1.00 (1.00, 2.00), while the ‘Sense of Time’ subscale had the lowest score at 0.00 (0.00, 1.00). See Table 2 for details.

**TABLE 2:** Death anxiety scores for community‐dwelling older adults.

Item	Mean	Score M (P25, P75)
Death anxiety score	4.93	5.00 (3.00, 6.00)
Emotions dimension	1.58	1.00 (1.00, 2.00)
Stress and suffering dimension	1.46	1.00 (1.00, 2.00)
Cognition dimension	1.39	1.00 (1.00, 2.00)
Sense of time dimension	0.50	0.00 (0.00, 1.00)

### 2.4 Scores on Death Education Needs Among Community‐Dwelling Older Adults

The total score for death education needs was 39.00 (34.00, 44.00). Among the items, ‘Do you believe that receiving death education is beneficial to the mental health of older adults?’ received the highest score of 4.00 (3.00, 5.00); whereas ‘Do you believe that everyone should receive education about death?’ received a relatively lower score of 4.00 (3.00, 4.00). See Table 3 for details.

**TABLE 3:** Scores for each item on the death education needs of community‐dwelling older adults.

Item	Mean	Score M (P25, P75)
Do you think community‐dwelling older adults should receive death education?	3.93	4.00 (3.00, 5.00)
Do you think older adults with chronic diseases should receive death education?	3.79	4.00 (3.00, 4.00)
Do you think death education would be very helpful for community‐dwelling older adults?	3.84	4.00 (3.00, 5.00)
Do you believe that death education can help older adults develop a correct understanding of death?	3.84	4.00 (3.00, 5.00)
Do you believe that conducting death education activities can help older adults gain a deeper understanding of death?	3.86	4.00 (3.00, 5.00)
Do you believe that death education can help older adults reduce their anxiety and fear of death?	3.84	4.00 (3.00, 5.00)
Do you believe that receiving death education contributes to the mental health of older adults?	3.96	4.00 (3.00, 5.00)
Would you like to receive more information about death education?	3.80	4.00 (3.00, 4.00)
Do you believe that death education should be taken seriously by the general public?	3.89	4.00 (3.00, 5.00)
Do you believe everyone should receive education about death?	3.79	4.00 (3.00, 4.00)
Total score	38.53	39.00 (34.00, 44.00)

### 2.5 Association Between Death Anxiety and Death Education Needs

Spearman correlation showed a weak positive association between death anxiety and death education needs (*r* = 0.122, *p* = 0.047). Among these, the ‘Stress and Suffering’ dimension showed the strongest correlation with the need for death education (*r* = 0.151, *p* = 0.014). See Table 4 for details.

**TABLE 4:** Correlation analysis of death anxiety and the need for death education among community‐dwelling older adults.

Dimension	Death education needs score	
	Spearman *r*	*p*
Emotional	0.079	0.197
Stress and suffering	0.151^a^	0.014
Cognitive	−0.002	0.974
Sense of time	0.040	0.519
Death anxiety score	0.122^a^	0.047

^a^ The correlation is significant at the 0.05 level (two‐tailed).

### 2.6 Comparison of Death Education Need Scores Among Older Adults in Communities With Different Characteristics

Univariate analysis shows that there are statistically significant differences in death education need scores among older adults with different educational levels (*H* = 8.139, *p* = 0.043) and monthly incomes (*H* = 18.095, *p* = 0.000). Older adults with a college degree or higher and a monthly income of ≥ 4000 yuan had higher demand scores. Gender, age, religious beliefs, marital status, type of illness, life‐threatening events, bereavement experiences and prior exposure to death education had no significant impact on death education demand scores; see Table 5 for details.

**TABLE 5:** Death education need scores among community‐dwelling older adults by characteristics.

Category	Subgroup	Mean	Score	Statistic	*p*
Gender	Male	38.33	38.00 (34.00, 45.00)	−0.191	0.849
	Female	38.64	39.00 (34.00, 43.75)		
Age	60–65	38.22	38.00 (34.00, 41.00)	1.565	0.457
	66–70	38.64	40.00 (32.75, 47.00)		
	71 and above	39.39	39.50 (34.50, 46.00)		
Educational level	Elementary School and below	37.63	36.00 (34.00, 41.00)	8.139	0.043
	Junior High School	37.76	38.00 (33.00, 42.00)		
	High school	38.26	38.00 (34.00, 43.50)		
	College or higher	40.94	41.00 (35.50, 48.50)		
Religious affiliation	Yes	39.11	37.50 (34.00, 44.75)	−0.063	0.95
	No	38.46	39.00 (34.00, 44.00)		
Monthly income	≤ 2000	37.50	37.50 (33.75, 41.00)	18.095	0.000
	2000–2999	37.04	37.00 (33.50, 40.50)		
	3000–3999	39.15	38.50 (34.00, 46.00)		
	≥ 4000	40.98	41.00 (38.25, 49.00)		
Marital status	Unmarried	32.75	31.50 (29.50, 37.25)	3.870	0.276
	Married with spouse alive	38.53	38.00 (34.00, 44.00)		
	Divorced	38.44	39.00 (34.50, 40.00)		
	Widowed	39.38	39.00 (36.00, 45.50)		
Health conditions	Chronic illness	38.59	39.00 (34.00, 43.00)	1.693	0.638
	Malignant tumour	37.63	38.50 (32.50, 44.75)		
	Traumatic illness	41.71	40.00 (36.00, 50.00)		
	No	38.42	38.00 (34.00, 44.50)		
Life‐threatening event	Yes	38.27	38.00 (34.25, 41.00)	−0.141	0.888
	No	38.59	39.00 (34.00, 45.00)		
Experience of bereavement	Yes	38.25	38.00 (34.00, 42.00)	−0.424	0.672
	No	38.94	39.00 (33.25, 45.00)		
Exposure to death education	Yes	38.54	37.50 (34.25, 46.75)	−0.175	0.861
	No	38.53	39.00 (34.00, 44.00)		

### 2.7 Multiple Linear Regression Analysis of Factors Associated With Death Education Needs

A multivariable linear regression model was fitted with educational level, monthly income, age, health conditions, bereavement experience and marital status. Educational level and monthly income were entered as categorical dummy variables; age was entered as a continuous variable. The overall model was marginally non‐significant (*F*(10, 256) = 1.72, *p* = 0.076) and explained 6.3% of the variance in death education needs (*R* ^2^ = 0.063; adjusted *R* ^2^ = 0.027). Compared with the reference category (monthly income ≤ 2000 yuan), the ≥ 4000 yuan category was associated with higher death education needs (*B* = 3.19, 95% CI 0.29–6.09, *p* = 0.031). Other monthly income categories, all educational level categories, health conditions, bereavement experience, marital status and age were not independently associated with death education needs. No multicollinearity was observed (all VIF < 5). Complete results are shown in Table 6.

**TABLE 6:** Multiple linear regression analysis of factors associated with death education needs among community‐dwelling older adults.

Variable	*B*	95% CI	*p*	VIF
Constant	33.89	27.81, 39.96	0.000	—
Education: junior high vs. elementary and below	−0.17	−3.22, 2.88	0.914	2.77
Education: high school vs. elementary and below	−0.25	−3.38, 2.88	0.874	3.35
Education: college/university and above vs. elementary and below	1.02	−2.79, 4.84	0.598	3.09
Monthly income: 2000–2999 vs. ≤ 2000	−0.25	−2.57, 2.08	0.836	1.52
Monthly income: 3000–3999 vs. ≤ 2000	1.72	−0.99, 4.43	0.213	1.64
Monthly income: ≥ 4000 vs. ≤ 2000	3.19	0.29, 6.09	0.031	2.18
Health status	0.01	−1.76, 1.78	0.991	1.11
Age	−0.01	−1.23, 1.21	0.986	1.10
Experience of bereavement	1.35	−0.46, 3.17	0.144	1.13
Marital status	0.76	−0.58, 2.10	0.264	1.09

*Note:* Model fit: *F*(10, 256) = 1.72, *p* = 0.076; *R* ^2^ = 0.063; adjusted *R* ^2^ = 0.027.

Linearity and homoscedasticity were examined using a residual‐versus‐fitted plot. The residuals were randomly distributed around zero without an obvious systematic pattern or funnel shape. The histogram and normal P–P plot of standardized residuals indicated that residuals were approximately normally distributed (mean ≈ 0; SD = 0.98). No influential observations were identified (maximum Cook's distance = 0.035; maximum leverage = 0.082). No multicollinearity was observed (all VIF < 5; maximum condition index = 19.86). These results suggest that the assumptions of linear regression were adequately met.

Sensitivity power analysis. A sensitivity power analysis using G*Power 3.1.9.2 for multiple linear regression with two predictors, *α* = 0.05, power = 0.95 and *N* = 267 indicated a minimum detectable effect size of *f* ^2^ = 0.059 (*R* ^2^ ≈ 5.5%). This indicates that the sample size was sufficient to detect moderate‐to‐small effects with high power. However, the final model explained only 6.3% of the variance (adjusted *R* ^2^ = 0.027), indicating limited power for very small effects. This limitation is acknowledged in the Section 5.

A sensitivity analysis was conducted by adding survey mode (online vs. face‐to‐face) to the main multivariable model. The overall model showed a trend towards significance (*F*(11, 255) = 1.68, *p* = 0.078; *R* ^2^ = 0.068; adjusted *R* ^2^ = 0.027). The ≥ 4000 yuan monthly income category remained independently associated with higher death education needs (*B* = 3.05, 95% CI 0.14 to 5.96, *p* = 0.040). Survey mode was not associated with death education needs (*B* = −1.22, 95% CI −3.36 to 0.92, *p* = 0.264). Other variables were not independently associated with needs. No multicollinearity was observed (all VIF < 5). These results suggest that the main findings were robust to survey mode. Complete results are shown in Table 7.

**TABLE 7:** Sensitivity analysis: multiple linear regression of death education needs controlling for survey mode.

Variable	*B*	95% CI	*p*	VIF
Constant	34.59	28.39, 40.78	0.000	—
Monthly income: ≥ 4000 vs. ≤ 2000	3.05	0.14, 5.96	0.040	2.19
Survey mode: online vs. face‐to‐face	−1.22	−3.36, 0.92	0.264	1.17
Other main‐model variables	Adjusted but not shown	—	—	—

*Note:* Model fit: *F*(11, 255) = 1.68, *p* = 0.078; *R* ^2^ = 0.068; adjusted *R* ^2^ = 0.027. Reference categories: monthly income = ≤ 2000 yuan; survey mode = face‐to‐face; health status = no chronic disease. Other main‐model variables (education, age, marital status, bereavement experience) were adjusted for but are not shown because they were already reported in Table 6.

## 3 Discussion

### 3.1 Death Anxiety Scores Among Community‐Dwelling Older Adults

In this study, the death anxiety score among community‐dwelling older adults was 5.00 (3.00, 6.00), with the emotional subscale showing the highest item‐level scores. This result is broadly consistent with relevant domestic studies. A survey by Zhang et al. (2025) of 559 community‐dwelling older adults showed that the average death anxiety score was 5.48 ± 3.64 for men and 6.70 ± 3.70 for women. The slightly lower score in this study may be attributable to the fact that the participants were predominantly relatively young older adults with good health and stable living conditions. Research by Kulakçı Altıntaş and Ercan Şahin (2025) further confirms that death anxiety levels among community‐dwelling older adults are closely associated with depressive symptoms and attitudes towards aging; older adults in better health often exhibit lower levels of death anxiety. Furthermore, the traditional cultural taboo against discussing death may lead older adults to avoid the topic, thereby influencing their reported scores on the scale (Peng et al. 2026).

### 3.2 Low Exposure to Death Education and Perceived Value

In this study, as many as 91.0% of community‐dwelling older adults reported never having been exposed to death education. This finding is consistent with the broader characterization of death education in China as sporadic and marginalized. At the same time, results indicated generally favourable attitudes towards the value and social importance of death education, with relatively higher scores on items such as ‘contributes to mental health’ and ‘helps develop a correct understanding of death’. These responses reflect perceived benefits and attitudes. A survey by Lei et al. (2022) of 683 community‐dwelling older adults in Chongqing found that community‐dwelling older adults have a high level of demand for death education, and that this demand is influenced by various factors such as monthly income and marital status. Our study suggests that high monthly income is associated with the need for end‐of‐life education, while education level is significant only in the univariate and does not remain independent in the multivariate model. Therefore, we do not recommend designing tiered death education programmes based on educational level at this stage. Any income‐related hypothesis‐generating should also be considered provisional. Future studies with stronger designs are needed before education or income‐based stratification can be used to guide intervention design.

### 3.3 Death Anxiety and Death Education Needs Were Weakly Positively Associated

A weak positive association was observed between death anxiety and death education needs, with the ‘stress and suffering’ dimension showing the strongest association among the subscales. This finding is consistent with the possibility that death‐related emotional experience may accompany greater interest in death education; previous research has examined psychological factors related to death anxiety, such as attachment style and inner peace, but these mechanisms were not tested in the present study (El‐Ashry et al. 2024). Death anxiety should therefore be regarded as one of many possible factors related to perceived death education needs. A meta‐analysis by Korean scholars Kim and Park systematically evaluated 11 death education intervention studies. The results showed that death education can significantly reduce death anxiety among older adults and improve their attitudes towards death, providing solid evidence‐based support for the effectiveness of death education (Kim and Park 2022). Because the ‘stress and suffering’ dimension showed the strongest correlation with needs, future studies may examine whether emotional support and coping strategies for illness and end‐of‐life situations are acceptable components of death education for community‐dwelling older adults. However, the present cross‐sectional data cannot establish program objectives or causal pathways.

### 3.4 Analysis of Community‐Dwelling Older Adults' Preferences Regarding Methods of Death Education

Among the methods of death education preferred by community‐dwelling older adults, ‘lectures’ accounted for the highest proportion. As a traditional form of health education, lectures are more readily accepted by older adults due to their authority, systematic nature and group setting. Second, watching films may gently and gradually prompt older adults to reflect on death and lower psychological defences; whether this format is acceptable or effective as an entry point should be tested in future studies. Research by Xie et al. (2024) confirms that narrative‐based life education programmes, grounded in the ‘knowledge‐attitude‐practice’ model, demonstrate good feasibility and acceptability among community‐dwelling older adults, significantly reducing their fear of death and enhancing their sense of life's meaning. Reading and group discussions, meanwhile, reflect some older adults' willingness to engage in deep thinking and share experiences, suggesting that future research could examine whether educational content should integrate cognitive and emotional dimensions. It is worth noting that the selection rates for experiential education methods such as ‘simulated farewells’ and ‘field trips’ were relatively low, which may be related to the traditional cultural taboo against discussing death and fear of death‐related scenarios.

These preference data may help generate hypotheses for future research. For example, future studies could test whether specialized lectures, film screenings and group discussions are acceptable and effective formats. Experiential methods such as simulated farewells should be examined cautiously because of their lower selection rates in this sample.

### 3.5 Income as a Correlate of Perceived Needs

The finding that only the highest monthly income category (≥ 4000 yuan) was associated with higher perceived death education needs in the main model is consistent with some prior evidence. Lei et al. identified monthly income as a significant factor influencing death education demand among community‐dwelling older adults in Chongqing. Higher income may facilitate access to information, participation in community activities and engagement with health‐related services, thereby shaping both awareness of and receptivity to death education. However, this association is unstable across different models: when health status and bereavement experience are added, the effect weakens to non‐significance, suggesting the apparent effect of income may be confounded by other socioeconomic and health‐related factors.

## 4 Implications for Nursing Practice

In this study, some older adults avoided direct death‐related questions. Nurses should avoid directly discussing death and instead use softer questions, listen and try to understand the person's needs. If the older adult still avoids the topic, do not push. Other older adults were calm and open; they wanted to know how diseases progress, how to adjust and how to cope near the end of life. Nurses can explain palliative care or advance care planning when needed. Some older adults may find death‐related information helpful, but whether death education relieves anxiety or improves quality of life was not examined in this study. The Death Education Needs Scale measures attitudes towards death education, not individual unmet needs. As this was only a cross‐sectional study, further research is needed to validate its application and specific models.

## 5 Limitations

This study has several limitations. First, it just used a cross‐sectional convenience sample from one city in western China, so causal inference and generalizability are limited. Second, the regression model explained only 6.3% of the variance and may be underpowered for very small effects. Third, the adapted Death Education Needs Scale showed only preliminary internal‐consistency and item‐structure evidence; no confirmatory factor analysis, test–retest reliability, criterion validity or independent cross‐validation was performed. Fourth, face‐to‐face administration of sensitive death‐related questions may have introduced interviewer effects and social‐desirability bias, with some participants possibly underreporting death anxiety or overreporting favourable attitudes. Fifth, although no sensitivity difference was found for survey mode, the online sample was small, and residual confounding by mode cannot be excluded. These findings are therefore preliminary and descriptive, and should be treated as hypothesis‐generating only.

## 6 Conclusion

This study provides regional evidence on death anxiety and death education needs among community‐dwelling older adults in western China. Exposure to death education was low, while perceived value was generally favourable. Death anxiety was weakly positively associated with death education needs, and only the highest monthly income category was associated with needs. The adapted scale requires further validation. These findings are descriptive and hypothesis‐generating; larger longitudinal or intervention studies are needed before they can guide practice or policy.

## Author Contributions

**Yan Wang:** conceptualization, methodology, data curation, formal analysis, investigation, writing – original draft, writing – review and editing. **Xiaolan Guo:** conceptualization, methodology, supervision, project administration, validation. **Liqun Huang:** conceptualization, methodology, supervision, funding acquisition, visualization, project administration, resources, writing – original draft, investigation. **Sha Li:** investigation, supervision, methodology, visualization. **Yuchen Wang:** methodology, visualization, investigation, software, data curation, supervision.

## Funding

This work was supported by (1) Scientific Research Program Funded by Education Department of Shaanxi Provincial Government (Program No. 23JZ034) and (2) University‐level scientific research project of Shaanxi University of Chinese Medicine (Program No. 2020GP11).

## Disclosure

The authors declare that no generative AI or AI‐assisted technologies were used during the preparation of this manuscript.

## Ethics Statement

This study was approved by the Ethics Committee of The Second Affiliated Hospital of Shaanxi University of Chinese Medicine (approval no. LW2026002‐1; approval date: 2026.2.2). Approval was obtained before participant recruitment began.

## Conflicts of Interest

The authors declare no conflicts of interest.

## Data Availability

The de‐identified data are available from the corresponding author upon reasonable request.

## References

[nop270962-bib-0001] Bao, J. , L. Zhou , G. Liu , et al. 2022. “Current State of Care for the Elderly in China in the Context of an Aging Population.” Bioscience Trends 16, no. 2: 107–118. 10.5582/bst.2022.01068 35431289

[nop270962-bib-0002] Chen, Q. 2023. “Research Progress on Death Education Needs of the Elderly at Home and Abroad.” Famous Doctor 8: 9–11.

[nop270962-bib-0003] El‐Ashry, A. M. , N. K. Abd El Fatah , and E. A. Abdelhamid . 2024. “Harmony Found: Serenity's Crucial Role in Alleviating Death Anxiety Through Attachment Styles Among Community‐Dwelling Older Adults.” Geriatric Nursing 58: 247–254. 10.1016/j.gerinurse.2024.05.035 38843753

[nop270962-bib-0004] Fortner, B. V. , and R. A. Neimeyer . 2007. “The Role of Age in Death Attitudes.” Death Studies 31, no. 6: 529–548.

[nop270962-bib-0005] Hu, Y. A. 2015. “Theoretical and Practical Analysis of General Education Moving Towards Life‐Orientation: Reflections Based on Elective Course of Life‐And‐Death Studies.” Theory and Practice of Contemporary Education 7, no. 10: 103–105.

[nop270962-bib-0006] Hu, Y. A. 2022. “Death Education: An Important Lesson to Enhance Life Awareness.” Educator 37: 9–11.

[nop270962-bib-0007] Huang, L. Q. , N. Ni , L. Zhang , et al. 2018. “Effectiveness of the ‘Four‐Step’ Death Education Model on Medical Students' Attitudes Towards Death.” New West 2: 132–133.

[nop270962-bib-0008] Jiang, S. , Z. Li , G. J. Lu , et al. 2019. “Hospice Care and Palliative Medicine: Conceptual Differentiation, Key Elements and Practical Application.” Medicine and Philosophy 40, no. 2: 37–42.

[nop270962-bib-0009] Kim, H. , and S. Park . 2022. “Effects of Death Education Programs Among Korean Aged in the Convergence Era: A Systematic Review and Meta‐Analysis.” Journal of Convergence Information Technology 12, no. 5: 71–82.

[nop270962-bib-0010] Kulakçı Altıntaş, H. , and N. Ercan Şahin . 2025. “Attitudes to Aging, Depression, and Death Anxiety Among Community‐Dwelling Older Adults: A Cross‐Sectional Correlational Study.” BMC Geriatrics 25, no. 1: 454. 10.1186/s12877-025-05839-3 PMC12219969 40604568

[nop270962-bib-0011] Lei, L. , H. Zhao , L. Ran , et al. 2022. “Influencing Factors of Attitudes Towards Death and Demands for Death Education Among Community‐Dwelling Chinese Older Adults: A Cross‐Sectional Study.” BMC Public Health 22, no. 1: 1242. 10.1186/s12889-022-13655-2 PMC9219144 35733112

[nop270962-bib-0012] Lin, X. , X. Y. Zhang , Y. J. Zhang , et al. 2024. “Study on the Current Status and Influencing Factors of Death Anxiety Among Rural Elderly in Sichuan.” Modern Preventive Medicine 51, no. 9: 1625–1629.

[nop270962-bib-0013] Liu, R. J. , M. H. Wang , Y. M. Yu , et al. 2022. “Community Home‐Based Hospice Care Service Model Under the Guidance of Tertiary Hospitals.” Acta Academiae Medicinae Sinicae 44, no. 5: 746–749. 10.3881/j.issn.1000-503X.15237 36325768

[nop270962-bib-0014] Peng, Y. , X. Zheng , Y. Huang , et al. 2026. “Perspectives on Death Among Rural Older Adults in Mainland China: A Qualitative Study.” Death Studies 50: 1311–1323. 10.1080/07481187.2025.2502986 40359231

[nop270962-bib-0015] Seckin, M. , R. Tiwana , D. Fry , and C. Bailey . 2025. “Key Themes and Approaches in Palliative and End‐of‐Life Care Education for the General Public: A Systematic Review.” BMC Palliative Care 24, no. 1: 219. 10.1186/s12904-025-01852-w PMC12333110 40775702

[nop270962-bib-0016] Smith, L. , and J. Harkness . 2011. “Death in Long‐Term Care Facilities: Attitudes and Reactions of Patients and Staff. A Qualitative Study.” European Geriatric Medicine 2, no. 1: 50–56.

[nop270962-bib-0017] Song, Z. M. , Y. Y. Wang , and W. W. Liu . 2019. “Current Status and Influencing Factors of Death Anxiety Among Community Residents.” Chinese General Practice 22, no. 28: 3510–3515.

[nop270962-bib-0018] Subiyanto, P. , and C. B. Susilo . 2024. “The Role of Community Nurses in Improving Public Health: Strategies and Implementation of Educational Programs.” Oshada 1, no. 4: 29–44.

[nop270962-bib-0019] Wang, L. , Y. Li , R. Zhao , et al. 2024. “Influencing Factors of Home Hospice Care Needs of the Older Adults With Chronic Diseases at the End of Life in China: A Cross‐Sectional Study.” Frontiers in Public Health 12: 1348214. 10.3389/fpubh.2024.1348214. PMC11163093 38859895

[nop270962-bib-0020] Xie, X. , L. Zhou , X. Zhang , H. Zou , Y. Lu , and H. Xiao . 2024. “Evaluation of a Knowledge‐Attitude‐Practice Model Based Narrative Life Education Program for Community‐Dwelling Older Adults: A Mixed‐Methods Feasibility Study.” BMC Geriatrics 24, no. 1: 547. 10.1186/s12877-024-05153-4 PMC11194897 38914955

[nop270962-bib-0021] Xu, B. H. , C. W. Hu , D. Q. Gu , et al. 2020. “Research Progress on Death Education for Patients.” Chinese Nursing Research 34, no. 12: 2170–2174.

[nop270962-bib-0022] Yan, S. H. 2002. Death Attitude and Life‐And‐Death Education Needs of Nursing College Students in Taiwan Region. Nanhua University.

[nop270962-bib-0023] Yang, H. 2011. Cross‐Cultural Adaptation and Application of the Death Anxiety Scale. Beijing University of Chinese Medicine.

[nop270962-bib-0024] Zhang, H. , Z. Xu , X. Zhai , et al. 2025. “Sex Differences in the Prevalence and Determinants of Death Anxiety Among Community‐Dwelling Older Adults in China: A Cross‐Sectional Study.” BMC Geriatrics 25, no. 1: 616. 10.1186/s12877-025-06297-7 PMC12341294 40796811

[nop270962-bib-0025] Zhang, Y. L. , L. Y. Wang , L. J. Xu , et al. 2022. “Preliminary Practice Outcomes of Demand‐Oriented Death Education for Community‐Dwelling Elderly.” Jiangsu Journal of Preventive Medicine 33, no. 1: 41–43, 62.

